# Pre‐existing heterologous T‐cell immunity and neoantigen immunogenicity

**DOI:** 10.1002/cti2.1111

**Published:** 2020-03-21

**Authors:** Qibin Leng, Marion Tarbe, Qi Long, Feng Wang

**Affiliations:** ^1^ Affiliated Cancer Hospital & Institute of Guangzhou Medical University State Key Laboratory of Respiratory Diseases, Guangzhou Medical University Guangzhou China; ^2^ The Joint Center for Infection and Immunity Guangzhou Women and Children's Medical Center Guangzhou Institute of Pediatrics Guangzhou Medical University Guangzhou China; ^3^ Institute Pasteur of Shanghai Chinese Academy of Science Shanghai China; ^4^ Department of Biostatistics, Epidemiology and Informatics Perelman School of Medicine University of Pennsylvania Philadelphia PA USA; ^5^ Department of Immunology and Microbiology Center for Microbiota and Immunological Diseases Shanghai General Hospital Shanghai Institute of Immunology Shanghai Jiao Tong University School of Medicine Shanghai China; ^6^ Research Center of Translational Medicine Shanghai Children's Hospital Shanghai Jiao Tong University School of Medicine Shanghai China

**Keywords:** cross‐reactivity, heterologous immunity, immunogenicity, microbial exposure, neoantigen, T cells

## Abstract

Neoantigens are tumor‐specific mutated proteins that are exempt from central tolerance and are therefore capable of efficiently eliciting effective T‐cell responses. The identification of immunogenic neoantigens in tumor‐specific mutated proteins has promising clinical implications for cancer immunotherapy. However, the factors that may contribute to neoantigen immunogenicity are not yet fully understood. Through molecular mimicry of antigens arising during cancer progression, the gut microbiota and previously encountered pathogens potentially have profound impacts on T‐cell responses to previously unencountered tumor neoantigens. Here, we review the characteristics of immunogenic neoantigens and how host exposure to microbes may affect T‐cell responses to neoantigens. We address the hypothesis that pre‐existing heterologous memory T‐cell immunity is a major factor that influences neoantigen immunogenicity in individual cancer patients. Accumulating data suggest that differences in individual histories of microbial exposure should be taken into account during the optimisation of algorithms that predict neoantigen immunogenicity.

## Graphical abstract

In this review, we discuss pre‐existing heterologous memory T‐cell immunity, which could be a major factor that influences neoantigen immunogenicity in individual cancer patients. Differences in individuals' histories of microbial exposure should be taken into account during the optimisation of algorithms that predict neoantigen immunogenicity.

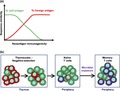

## Introduction

Most tumor‐associated antigens (TAAs) targeted in clinical applications are overexpressed self‐proteins, namely self‐antigens. Self‐antigens have low immunogenicity because the high‐affinity T cells that recognise these antigens are deleted through the mechanism of central tolerance during thymic development to avoid triggering autoimmunity. Low immunogenicity is one of the key reasons for the ineffectiveness of TAA‐based therapeutic antitumor vaccines. In contrast to low‐immunogenicity TAAs, neoantigens, which are generated during the degradation of mutated proteins, are promising anticancer vaccines. As they are considered to be foreign peptides by the immune system, they may be highly immunogenic and capable of eliciting strong T‐cell‐mediated immune responses.

Previously, a therapeutic MHC I‐restricted neoantigen vaccine was shown to elicit effective T‐cell immunity that led to tumor rejection in mice.^1^ More promisingly, two independent phase I clinical trials evaluating personalised neoantigen vaccines triggering both CD4 and CD8 T‐cell activation showed encouraging results in patients with melanoma.^2^, ^3^ Neoantigen vaccination has also been shown to effectively elicit T‐cell responses in patients with glioblastoma, which is classified as an immunologically cold tumor characterised by few infiltrating immune cells and a relatively low mutational burden.^4^ Furthermore, mounting evidence demonstrates that the load and quality of neoantigens are biomarkers for predicting the efficacy of checkpoint inhibitor‐based cancer immunotherapy.^5^, ^6^, ^7^ Therefore, the identification of immunogenic neoantigens has promising clinical implications for cancer immunotherapy.

Currently, the identification of candidate neoantigens is performed mainly with *in silico* human leucocyte antigen (HLA)‐binding prediction algorithms after pinpointing missense mutations in patient tumor tissue by exome sequencing. These neoantigen prediction algorithms are effective for the majority of HLA class I alleles but perform rather poorly for HLA class II alleles.^8^, ^9^, ^10^ Although novel algorithms based on mass spectrometry data have been developed to improve prediction of neoantigens presented by HLA class II alleles,^11^, ^12^, ^13^ it still remains unclear whether predicted neoantigen candidates are immunogenic. In fact, the frequency of immunogenic neoantigens among candidates is very low.^14^, ^15^ Besides MHC binding, other factor, such as the T‐cell repertoire of an individual patient, may be involved in the immunogenicity of neoantigen candidates. Evidently, the T‐cell repertoire is not only shaped by genes but also tuned by environmental factors, including viral or bacterial infections,^16^, ^17^, ^18^ vaccines^19^ and the gut microbiota.^20^

In this review, we discuss the characteristics of immunogenic peptides and then elucidate how the gut microbiota and other environmental microbes may affect neoantigen immunogenicity via cross‐reactive heterologous memory T‐cell immunity as a result of molecular mimicry among antigens. We propose that pre‐existing memory T‐cell immunity to these microbes is likely to be an immunoediting factor that sculpts tumors in individual patients by influencing neoantigen‐specific T‐cell responses.

## Neoantigen foreignness and similarities between neoantigens and microbial antigens

Although the factors that affect neoantigen immunogenicity have not been specifically addressed thus far and remain largely unknown, the features of neoantigens correlated with immunotherapeutic efficacy or disease prognosis have been described previously.^5^, ^6^, ^7^ A study by Snyder and colleagues showed that the neoantigens in cancer tissue samples from cytotoxic T‐lymphocyte‐associated antigen 4 (CTLA‐4) inhibitor‐treated patients who exhibited long‐term clinical benefit shared a number of tetrapeptide sequences that were completely absent in patients who received no or minimal benefit. The candidate neoantigens containing the shared tetrapeptides were homologous to many viral and bacterial antigens,^6^ indicating potential cross‐reactivity of T‐cell response to the neoantigens with microbial antigens. However, this tetrapeptide feature was not confirmed in a later analysis of human neoantigens identified in other studies.^10^, ^21^

Nevertheless, as the host TCR repertoire evolutionarily adjusts itself to detect pathogenic antigens, it is believed that neoantigens homologous to pathogenic antigens are more likely to be immunogenic than nonhomologous neoantigens.^5^, ^7^ Accordingly, a composite neoantigen quality model has been proposed to score the potential immunogenicity of neoantigen candidates on the basis of sequence homology with pathogenic antigens and the relative predicted HLA‐binding affinity of the neoepitope compared with that of the corresponding wild‐type peptide. Interestingly, this quality model is capable of discriminating between long‐ and short‐term survivors of pancreatic cancer. In other words, neoantigen quality serves as a predictive biomarker of survival in patients with pancreatic cancer.^7^ It was found that T‐cell clones in the peripheral blood could cross‐react with both neoantigens and predicted cross‐reactive microbial epitopes. Moreover, these clones were also present in primary tumors, leading to an improved antitumor immune response.^6^, ^7^ Collectively, these studies indicate that the sequence homology between a neoantigen and microbial peptide at least partially contributes to the immunogenicity of the neoantigen.

## Neoantigen immunogenicity and T‐cell recognition

One of the key features of T cells is their antigen specificity determined by the TCR. Although T‐cell recognition is very sensitive to a specific antigen,^22^ this process is also quite indiscriminate. When presented by an MHC complex, many different peptides, which generally have different MHC anchoring residues, have highly similar three‐dimensional shapes or sequence features that can be recognised by a single TCR. Thus, one T‐cell clone with a fixed TCR can recognise approximately one million peptide epitopes with distinct primary sequences.^23^, ^24^ Reciprocally, one peptide can be recognised by many TCR clones with diverse TCR sequences sharing conserved antigen‐binding motifs.^23^, ^25^, ^26^, ^27^, ^28^, ^29^, ^30^, ^31^ Moreover, several studies have revealed that TCR contact conservation in peptide antigens contributes to cross‐reactivity.^29^, ^32^, ^33^ One conserved anchoring amino acid associated with two or more conserved TCR‐contacting amino acids within a core nonamer is sufficient to enable two different MHC II‐binding peptides to cross‐react with the same TCR.^29^, ^34^ Similarly, an MHC I‐restricted CD8 T‐cell TCR can cross‐react with altered peptide antigens harbouring one or two substitutions in the TCR contact positions.^35^, ^36^, ^37^ Additionally, the conservation of a binding motif comprising only three amino acids in the central part of HLA‐A2 ligands has been shown to be sufficient to activate an autoreactive TCR clone.^31^, ^38^ The complexity of the interaction between a peptide/MHC complex and TCR can explain the discordant findings regarding tetrapeptides shared by neoantigens and microbial peptides: the shared tetrapeptides are neither necessarily required nor sufficient to determine the cross‐reactivity between neoantigens and microbial homologs.^6^, ^10^

However, studies have shown that homology among TCR‐contacting residues contributes to TCR cross‐reactivity between self‐peptides and microbial or environmental peptides.^29^, ^30^ The degree of similarity between a foreign peptide antigen and self‐antigen determines the number of precursor T cells specific for the foreign antigen. Thus, the higher the number of self‐antigens similar to a foreign peptide antigen, the smaller the foreign peptide‐specific T‐cell population will be as a result of increased clonal deletion in the thymus.^29^

**Figure 1:**
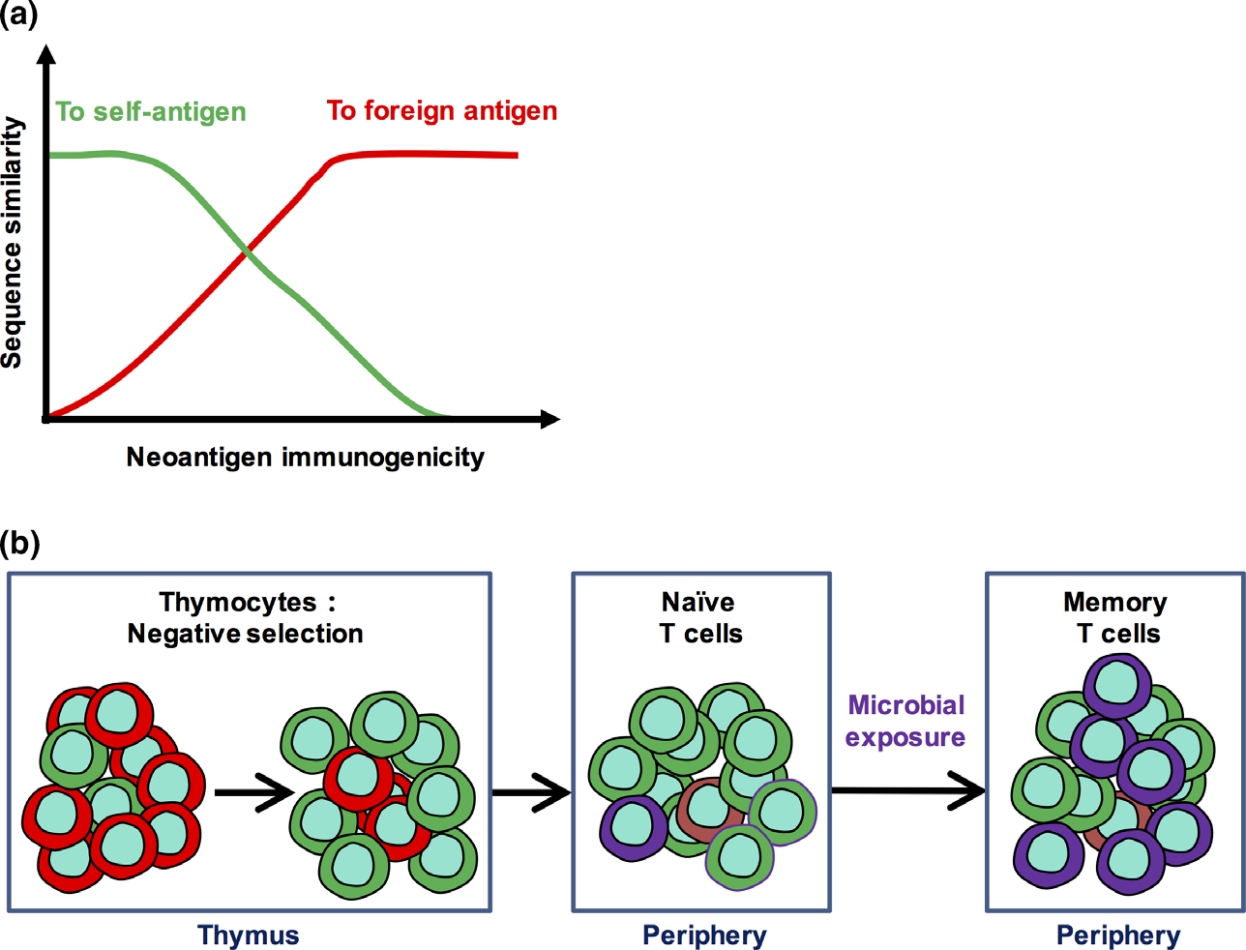
Central tolerance and microbial exposure both affect neoantigen immunogenicity. **(a)** Neoantigen immunogenicity is related to the degree of similarity between the amino acid (AA) sequence of the neoantigen and the AA sequences of both self‐antigens (green line) and foreign antigens (red line). AA sequence similarity can be characterised by the following features, such as peptide sequence homology, three dimensional shape, and hydrophobicity, charge and length of the side chain of the mutated amino acid. **(b)** Central tolerance deletes T cells (red) that strongly react with self‐antigens through the process of negative selection and spares T cells that mainly react with foreign antigens (green and purple). Microbial exposure increases the likelihood of neoantigen immunogenicity by expanding cross‐reactive heterologous memory T cells (purple).

Neoantigens are generated from mutated self‐antigens; thus, the degree of similarity in three‐dimensional shape between the TCR contact residues of a neoantigen and those of a self‐antigen determines the foreignness of the neoantigen. In other words, neoantigen immunogenicity is set by the degree of similarity between the TCR contacts of a neoantigen and those of both self‐antigens and foreign antigens (Figure 1a).

## Microbiota as a potential factor that influences T‐cell responses to neoantigens

Mammals have trillions of gut microorganisms, including predominantly bacteria but also fungi, archaea, viruses and protozoans. The microbiota coexists with the host and is highly related to both the development and function of the immune system. The interactions of defined microorganisms with their host can be highly contextual with the same microbe developing as a mutualist or parasite according to the nutritional, coinfection or genetic landscape of the host. As pathogens, the microorganisms of the microbiota can lead to infection and inflammation; as mutualists, they help the host digest food, act as a barrier for pathogen defence and regulate a variety of mucosal immune responses.^39^, ^40^

More deeply, commensal microorganisms are required for the maturation of the immune system, which ‘learns’ to distinguish commensal bacteria from pathogenic bacteria and tolerate these commensal microorganisms. The gut microbiota continues to influence the immune system in different ways. It has been shown to modulate neutrophil migration and function and to affect the differentiation of T‐cell populations into different types of helper cells (Th) or into regulatory T cells. Additionally, the gastrointestinal tract is one of the main entry sites for pathogens. In gastrointestinal infections, pathogens are captured by Peyer's patch‐resident M cells that allow pathogen‐derived antigens to be transferred across the epithelial cell layer and delivered to the peripheral immune system, specifically to antigen‐presenting cells such as dendritic cells (DCs), leading to the activation of T cells, and B cells, leading to the secretion of IgA antibodies. Interestingly, commensal antigens also use this mechanism to induce the production of low amounts of IgA antibodies through the modulation of their immunodominant epitopes to facilitate colonisation.^41^

Through exposure to various complex bacterial antigens, the immune system accurately adapts its innate and adaptive responses against self‐antigens and nonself antigens. Accumulating evidence shows the function of the gut microbiota in modulating both host carcinogenesis and antitumor immunity. The previously mentioned study of long‐term survivors of pancreatic cancer found that neoantigen‐specific T cells existed in the peripheral blood and that tumor‐infiltrating leucocytes cross‐reacted with both neoantigens and homologous microbial antigens.^7^ This intriguing result indicates that microbial exposure may shape T‐cell responses to cancer neoantigens by modulating T‐cell cross‐reactivity.

Gut commensal microbes that coexist innocuously with the host express the foreign antigens that the host is most frequently exposed to. Exposure to microbial antigens not only generates memory T cells specific for these antigens but also increases the number of T cells that cross‐react with previously unencountered antigens. Therefore, the gut microbiota potentially has a profound impact on the T‐cell response to previously unencountered tumor neoantigens arising during cancer progression or viral antigens acquired in a relatively late stage. A study by Su *et al*. showed that memory‐phenotype, human immunodeficiency virus (HIV) antigen‐specific T cells existed in the peripheral blood of HIV‐seronegative adults. In contrast, these memory‐phenotype cells were absent in umbilical cord blood at birth. Interestingly, these HIV‐specific T cells produced IFN‐γ and IL‐2 when stimulated with peptides from gut microbes, including human gut commensals such as *Ruminococcus*, Lachnospiraceae and *Bifidobacterium* species. Therefore, one plausible conclusion is that host exposure to the cross‐reactive antigens of diverse environmental or gut microbes results in the generation of memory T cells that can respond to unencountered antigens,^20^, ^42^ including both viral antigens and somatic mutation‐derived neoantigens.

The gut microbiota greatly influences both innate and adaptive immune responses. Recently, it was revealed that microbiome sequence similarity can increase or decrease the immunogenicity of MHC II‐restricted CD4 T‐cell epitopes.^43^ Inflammatory or tolerogenic influences tend to be associated with subsets of microbial genera; *Fusobacterium* is mostly related to inflammatory influences, and *Bacteroides* is mostly related to tolerogenic influences in humans.^43^ Interestingly, several studies have shown that specific genera of gut microbes are associated with the efficacy of checkpoint inhibitors. *Bifidobacterium* was found to be associated with the antitumor effects of programmed cell death protein 1 ligand 1 (PD‐L1)‐specific antibody therapy in a mouse model of melanoma. Oral administration of *Bifidobacterium* to recipient mice with an established tumor was shown to increase the tumor‐specific CD8 T‐cell response and reduce tumor growth.^44^ Similarly, *Bacteroides fragilis* and *Burkholderia cepacia*, which both belong to the genus *Bacteroides*, enhance the antitumor effect of anti‐CTLA‐4 antibodies in mice,^45^ while *Bifidobacterium* species ameliorate the gut immunopathology associated with CTLA‐4 blockade.^46^ In the context of programmed cell death protein 1 (PD‐1) blockade, *Akkermansia muciniphila* has been identified to be associated with the best clinical response in non‐small‐cell lung cancer (NSCLC) patients.^47^ Although the gut microbiota generally contributes to the efficacy of checkpoint inhibitors, it is important to note that the bacteria identified to modulate the efficacies of different checkpoint inhibitors are completely distinct in different studies. Nevertheless, the increases in antitumor effects are consistently attributed to memory CD4 T‐cell‐dependent mechanisms in both CTLA‐4 and PD‐1/PD‐L1 blockade therapies. Thus, it is speculated that molecular mimicry between gut microbiota epitopes and tumor neoantigens as a result of their antigenic similarities can account for the efficacy of immune checkpoint inhibitors.^45^

However, sequence similarity with a microbiota‐derived epitope does not guarantee that an epitope will have higher immunogenicity than an epitope without sequence similarity. It has been reported that CD4 T‐cell epitope immunogenicity tends to be associated with the nature of the microbiota that shares sequence similarity with the epitope.^43^ For example, in addition to sequence similarity, microbiota‐derived signals are able to polarise antigen‐primed CD4 T cells into distinct T helper subsets, contributing to distinct immune responses.^48^ Thus, future investigations should investigate how sequence similarity between neoantigens and microbiota‐derived antigens affects the T‐cell response and consider this impact in combination with other immune features of the microbe from which the microbial epitope is derived.

## Pre‐existing heterologous immunity affects subsequent T‐cell responses to unrelated antigens

Unlike mice that live in specific‐pathogen‐free environments, humans are constantly exposed to various microbes after birth and receive various microbial vaccines. A serological profiling study of human populations revealed that every adult was exposed to an average of 10 viral species and some adults were even exposed to 84 species.^49^ Microbial exposure or infection can have both short‐term and long‐term effects on the immune system, particularly transforming subsets of T cells into memory cells. Over time, memory T cells accumulate and become an important part of the T‐cell repertoire^42^, ^50^, ^51^; therefore, these cells influence immune responses against new challenges (Figure 1b).

It has been shown that prior exposure to a related or completely unrelated pathogen can alter the host immune response to a second heterologous pathogen. This phenomenon is named heterologous immunity. Memory T cells play a critical role in long‐term heterologous immunity.^52^, ^53^ Pre‐existing T‐cell memory enhances T‐cell responses to cross‐reactive epitopes, shaping the epitope dominance of secondary viral infections.^54^, ^55^, ^56^ Cross‐reactive memory T cells can contribute to protective immunity or immunopathology upon a secondary virus infection, most likely because of the enhancement of the T‐cell response. For example, a patient who has been infected with one of the four dengue virus serotypes will more likely develop severe haemorrhagic fever upon infection with a second serotype.^57^, ^58^ The activation of cross‐reactive memory CD8 T cells specific for influenza A virus has been found to be associated with acute infectious mononucleosis caused by Epstein–Barr virus (EBV).^59^ Because memory T cells respond to antigens earlier and more robustly than naive T cells, it is believed that the pre‐existence of cross‐reactive memory T cells may contribute to individual differences in the clinical outcomes of viral infections, such as hepatitis C virus (HCV), EBV and HIV infections in humans.^52^, ^60^

How pre‐existing heterologous immunity to viral and microbial pathogens affects neoantigen‐specific T‐cell responses has not yet been fully investigated. However, the previously mentioned study by Balachandran *et al*.^7^ found that some neoantigen‐specific T cells were cross‐reactive with homologous noncancer microbial antigens in the long‐term survivors of pancreatic cancer. Thus, it is plausible that pre‐existing heterologous immunity induced by prior microbial exposure in cancer patients likely influences T‐cell response to tumor neoantigens via the cross‐reactivity of homologous microbial epitopes. Consistent with the frequent exposure to common viral pathogens, such as human cytomegalovirus, human papillomavirus and hepatitis C virus, in humans,^49^ epitopes from these viruses were found to cross‐react with neoantigens in cancer patients^6^, ^7^ (Table 1). Interestingly, the frequencies of TCR repertoire recognising these common pathogens are higher because of the clonal expansion.^61^ Thus, it is most likely that the neoantigens that cross‐react with antigens of common pathogens elicit more robust T‐cell response as a result of recalling heterologous memory response than those neoantigens that are recognised by naïve T cells.

**Table 1:** Validated neoantigens that share homologs to microbial antigens

Wild‐type peptide	Neoantigen peptide	Cross‐reacting microbial peptide	HLA restriction	Microbial species	Reference
YLLGSSALT	YLL**E**SSALT	***SSA***KRKMDPD	HLA‐A*2402	Human cytomegalovirus	6
VGSSADILY	V**E**SSADILY	***SSA***KRKMDPD	HLA‐A*2402	Human cytomegalovirus	6
YFPEESSAL	Y**S**PEESSAL	***SSA***KRKMDPD	HLA‐A*2402	Human cytomegalovirus	6
GLERGGFTF	GLER**E**GFTF	A***L***K***REGFTF***	HLA‐A*03:01	*Burkholderia pseudomallei*	6
TKSPFEQHI	T**E**SPFEQHI	GVP***ESPF***SRT	NR	Hepatitis D virus	6
QEFENIKSS	QEFENIKS**Y**	***Q***R***F***H***NI***RGR	HLA‐A*C1203	Human papillomavirus	7
GIICLDCKL	GIICLD**Y**KL	TMGV***L***CLAI***L***	HLA‐A*A0201	Dengue virus	7
LSLMSTLGI	L**L**LMSTLGI	***LLM***G***TLGI***V	HLA‐A*A0201	Human papillomavirus	7
QTYQRMWNY	QTYQ**H**MWNY	AFWAK**H** ***MWN***F	HLA‐A*A0301	Hepatitis C virus	7
LPRQYWEEL	LPRQYWE**A**L	KLLPEG***YW***V	HLA‐A*B0702	***Francisella tularensis***	7
RPQGQRPAP	RPQGQRPA**L**	SPR***G***S***RP***SW	HLA‐A*B0702	Hepatitis C virus	7
RVRDIVPTL	RV**W**DIVPTL	KP***WD***V***VPT***V	HLA‐A*A0201	Dengue virus	7

Bold letters in neoantigens indicate mutated amino acids that are different from wild‐type peptide. The italic letters in microbial antigens indicate amino acids that are identical in neoantigen sequences. NR indicates no report on the HLA restriction.

HCMV IE‐1 protein contains MESSAKRKMDPDNPD, which shares tetrapeptide ESSA with neoantigens, YLLESSALT, VESSADILY and YSPEESSAL, whereas the peptide MESSAKRKM is predicted to be presented by HLA‐B*40:01, HLA‐B*40:02 or HLA‐B*40:03 when analysed with IEDB algorithm. We show the validated HLA‐A*2402‐restricted HCMV epitope, ***SSA***KRKMDPD.^64^

## Conclusion and perspectives

In all, evidence supports the possibility that microbial exposure can shape neoantigen‐specific T‐cell responses through TCR‐mediated cross‐reactivity (Table 2). It remains largely unknown how microbial exposure influences host T‐cell response to cross‐reactive neoantigen. As exposure to microbes generates memory T cells and memory T cells respond to antigen more quickly and strongly, microbial exposure likely enhances neoantigen immunogenicity by eliciting heterologous memory T‐cell immunity in hosts. However, it cannot rule out the possibility that the types of exposed microbes are also critical for affecting host T‐cell response to cross‐reactive neoantigens. In other words, are the neoantigens that are cross‐reactive to pathogenic microbes more immunogenic? Are the neoantigens that are cross‐reactive to tolerant commensal microbes less immunogenic? Thus, it merits further investigating how microbial exposure affects neoantigen immunogenicity through heterologous cross‐reactive memory T‐cell immunity. Furthermore, heterologous immunity, as an immune editor,^62^ likely sculpts the landscape of neoantigens, thus affecting tumor immunogenicity in individual patients. Since the TCR repertoire is a central component that determines the specificity of heterologous immunity, recent progress in methods for both TCR sequencing and functional gene expression analysis at the single T‐cell level will shed light on the mechanism underlying this important issue.^63^

**Table 2:** The evidence for the possibility that microbial exposure can shape mutation‐derived neoantigen‐specific T‐cell responses through TCR‐mediated cross‐reactivity

Supporting evidence	Reference
(1) Direct cross‐reactivity of neoantigens to microbial antigens	6, 7
(2) Existence of memory‐like human T cells that respond to unexposed antigens	46
(3) Heterologous memory T cells affect the subsequent response to cross‐reactive antigens.	56, 57, 58
(4) The observations that microbiome can influence cancer development and responsiveness to immunotherapies	48, 49, 50, 51

Given that gut microbiota and environmental bacterial exposure histories vary among individuals in different countries, pre‐existing heterologous memory T‐cell immunity may also vary among individual cancer patients. The T‐cell immune repertoire and function shaped by regional differences in the gut microbiota, environmental bacteria and endemic infectious agents should be investigated in the process of determining the quality or immunogenicity of a neoantigen. In general, we propose that the degree of similarity between microbial antigens and self‐antigens can be considered to be an immunogenicity parameter for neoantigen prediction.

## Conflict of interest

The authors declare no conflict of interest.
